# Learning health equity in higher education—results from a European pilot study

**DOI:** 10.1186/s12909-026-08979-1

**Published:** 2026-03-11

**Authors:** Ingrid Onarheim Spjeldnæs, Gwendolijn Boonekamp, Ana Belén Subirón-Valera, Cia Törnblom, Anu Nyberg, Beatriz Rodríguez-Roca, Joost van Wijchen, Isabel Antón-Solanas, Maria Nordheim Alme

**Affiliations:** 1 Faculty of Health and Social Sciences, Western Norway University of Applied Sciences, Post Box 7030, Bergen, N-5020 Norway; 2 School of Sports and Exercise, HAN University of Applied Sciences, Nijmegen, Netherlands; 3 Faculty of Health Sciences, University of Zaragoza, Zaragoza, Spain; 4 School of Engineering, Culture and Wellbeing, Arcada University of Applied Sciences, Helsinki, Finland; 5 Academy of Business and Health Care, Arcada University of Applied Sciences, Helsinki, Finland

**Keywords:** Health equity, Education, Transforming pedagogy, Professional development

## Abstract

Health equity is a critical concern in global health and a growing priority in higher education. This paper presents findings from a European pilot study conducted within the project of Health Equity Through Education for a Sustainable Future (HEQED), which aimed to explore and develop strategies for fostering students’ understanding of health equity and their capacity to act on this knowledge in professional contexts. The study involved 264 participants from four higher education institutions across Europe, who participated in a structured learning activity centred on real-world cases illustrating systemic health inequities. Using a post-activity survey and thematic analysis of open-ended responses, this study aims to examine students’ perceptions of the relevance, impact, and pedagogical value of the intervention. Responses indicated that students found the topic highly relevant and the learning experience both meaningful and transformative. The activity promoted critical reflection, empathy, and awareness of important aspect related to health equity, while also challenging students to consider their professional responsibilities in addressing inequities. The results were interpreted and understood in terms of the following three analytic themes; 1) confronting cases, 2) reflexive practise, and 3) professional development. In conclusion, educational triggers can promote reflection and competence in complex issues, such as health equity. For further understanding, long-term impacts should be explored.

**Supplementary Information:**

The online version contains supplementary material available at 10.1186/s12909-026-08979-1.

## Background and introduction

Health equity refers to the absence of avoidable and unjust differences in health between groups defined by social, economic, demographic, or geographic conditions [13]. Health equity is widely recognised as a normative objective, for which various strategies have been developed to assess its outcomes. These include the use of health impact assessments, community health planning, policy evaluation frameworks and guidelines [22, 34]. These health inequities are not primarily the result of personal choices or genetics, but stem from structural factors that shape the distribution of resources and opportunities across society. Research by Marmot and colleagues [39, 40] has demonstrated that access to education, housing, income security, employment, safe environments, and healthcare are key determinants of health. These elements create a social gradient in which individuals who are lower on the socioeconomic ladder, experience poorer health and shorter lives.

Addressing health inequities requires more than improving aggregated health outcomes; it demands dismantling the systemic conditions that produce disparities. This work cannot be confined to the health sector alone. The health equity measurement framework [23, 51] and European initiatives [25] underline the need for multisectoral efforts. Higher education, as a key societal institution, holds significant, yet often untapped, potential to contribute to this effort. Higher education has a dual responsibility in the context of health equity. It must foster awareness among students about how their own health opportunities are shaped by social determinants and, at the same time, prepare students to act ethically and effectively in their future professional roles to address these inequities. Accordingly, the paper at hand aims to contribute to this field of knowledge by addressing the concept of health equity in a higher education learning context by exploring student’s perceptions of learning about this concept.

Fostering awareness among students resonates strongly with Sen’s capability approach, which defines equity as the real freedom to lead a life one has reason to value [50]. Education not only enhances individual capabilities, but also develops agency, the capacity to act on one’s values and effect social change [47]. The citizen scholar model [5] offers a pedagogical orientation aligned with these goals. It calls for a radical rethinking of university education, emphasizing the development of engaged graduates who are capable of critical thinking, empathy, and problem-solving [10]. This requires moving beyond content heavy instruction toward interactive, student-centred pedagogies that prioritize real-world engagement and civic responsibility [9]. To meet this emancipative and transformative potential, education must go beyond content delivery [1, 7]. As Velardo [56] emphasizes, students may enter higher education with an individualistic understanding of health disparities, shaped by neoliberal discourses of personal responsibility. We follow [24, 31] when they emphasise that higher education must actively disrupt these assumptions through pedagogies that foster social awareness, and empathetic and knowledge-based understanding of how structural factors like racism, poverty, and exclusion shape health.

We furthermore acknowledge how awareness of health inequities, while essential, is not sufficient on its own. Higher education must also cultivate students’ capacity to act upon this understanding. Drawing on critical health literacy and Paulo Freire’s concept of critical consciousness [27], pedagogical approaches should empower students to question dominant ideologies, engage in collective inquiry, and develop the agency to advocate for change [37]. This involves equipping learners with practical tools, such as advocacy skills, problem-solving strategies and an orientation toward ethical action, through interactive and inclusive methods like scenario-based learning, civic engagement projects, and reflective group dialogue [35]. Such approaches align with the principles of epistemic liberation, which encourage exploration of diverse and often marginalised ways of knowing, and support transformative learning by challenging students’ assumptions and reshaping their engagement with the world [36, 42]. The capability approach [45, 50] further reinforces this by fostering empathy, critical thinking, and value-driven action, while salutogenic theory [2] situates learners as capable of navigating complexity through a sense of coherence, comprising comprehensibility, manageability, and meaningfulness. Together, these theoretical perspectives converge to support pedagogical strategies such as case-based learning, participatory simulations, and role-plays. These methods immerse students in real-world complexity and cultivate their ethical and civic imagination, preparing them not only to understand the structural dimensions of health inequities but to address them with resilience, purpose, and a commitment to social justice.

Through the Health Equity Through Education for a Sustainable Future (HEQED) project [32], which is a consortium of four European Universities and one non-governmental organisation (NGO), we initiated a joint effort to build competence by operationalising health equity within educational programs. The HEQED project aims to explore how health equity can be learned and taught within higher education settings by engaging students and educators across disciplines in transformative learning about health equity and develop pedagogical strategies that foster both critical awareness and agency. One part of the project is to develop learning activities purposefully designed to prompt students to reflect on the concept of health equity, engage empathetically with its real-world implications, and begin to envision their own professional responsibility in addressing systemic health disparities. The activities are designed for context dependent adaptions for meaningful use across institutions and study programmes. The activities use a “trigger” [6, 8], a case or scenario intended to serve as a shared entry point into the complexities of health inequity. This trigger is a complex context-specific scenario involving structural determinants of health with the intention to spark critical questioning, generate discussion, and create an emotional and intellectual entry point into the topic. By anchoring the session in a realistic narrative, the aim is to challenge students’ assumptions, expose them to the lived realities of inequity, and cultivate a learning environment where ethical and social dimensions of health could be explored collaboratively.

The learning activities are part of a health equity framework that is being developed within the HEQED consortium, and operationalised upon the basis of the following five conceptual ‘lenses’: *social justice, participation, human rights, planetary well-being, and ontological security* (https://heqed.org/).* The social justice lens* emphasises fairness in the distribution of resources, opportunities, and health outcomes, highlighting how intersecting social factors such as gender, race, and age contribute to structural inequities [16, 48]. Rooted in the capability approach [45, 50], it views health as both a right and a means to pursue a meaningful life, underscoring the interdependence of individual, collective, and planetary well-being. Complementing this, *the human rights lens* affirms the inherent dignity and equal worth of all individuals, advocating for equitable access to health and protection from discrimination. It draws on international frameworks to promote systemic accountability and ethical responsibility [4, 29]. *The ontological security lens* adds a psychosocial dimension, focusing on individuals’ need for identity coherence, stability, and continuity. It highlights how secure environments, both material and relational, enable people to develop agency, resilience, and a sense of meaning [30]. *The planetary health len*s situates human well-being within the broader ecological context, recognising the interconnectedness of environmental sustainability and social equity. It calls for a redefinition of progress that respects ecological boundaries and promotes long-term resilience for both human and non-human systems [33, 44]. Finally, *the participation lens* foregrounds inclusive, democratic engagement as a foundation for equity and social cohesion. It emphasises the importance of empowering individuals and communities to influence decisions that affect their lives, thereby fostering agency, belonging, and collective responsibility [14, 26]. Together, these lenses offer a multidimensional framework for understanding and advancing health equity. They highlight the need for integrated, justice-oriented approaches that address structural determinants, promote inclusive participation, and support both human and planetary flourishing. As part of the HEQED framework, health equity case studies, or learning activities, have been developed for use in both classroom and individual learning contexts. Each case is supported by a set of guiding questions designed to scaffold students’ progression from understanding and empathy toward agency and action. The use the conceptual lenses enables students to interrogate each case from multiple perspectives, supporting critical reflection and preparing them to contribute meaningfully to advancing health equity in their future professional roles.

This pilot study explores how equity-oriented learning activities can support the development of critical awareness and professional agency among higher education students engaging with the topic of health equity. By placing student perspectives at the centre stage, the research highlights the transformative potential of education as a driver of ethical reflection and civic responsibility in the pursuit of more equitable health outcomes. *The study aims to explore student’s perceptions of the relevance, impact, and pedagogical value of the health equity learning situations.*

## Methods

### Learning activities

The learning activities were designed as case-based triggers to stimulate students’ critical reflection, drawing on principles of Problem-Based Learning (PBL). PBL encourages active, student-centred inquiry grounded in real-world complexity [8] making it particularly well-suited for exploring issues such as health equity. In this pilot, the following two activities were used: the “Alejandra and Maria” story and the “Dressing room”. The former presents a scenario involving a mother suffering from drug addiction and her newborn daughter, while the latter invites students to “dress” or “undress” a persona with various socioeconomic attributes. Both were designed to prompt reflections on structural determinants of health (see details in Supplementary Material 1). While structured instructions accompany each activity, they are intended as flexible guides rather than rigid protocols. Adaptation to the specific learning context is encouraged, such as tailoring the activity in relation to the particular educational programme and level, time constraints, and group size. These activities are not designed as linear exercises, but as pedagogical tools grounded in real-life scenarios that are informed by one or more health equity lenses (the lenses described in the "Background and introduction" section).

### Recruitment and participants

Participants were drawn from health and social science programmes from the HEQED partner universities. Educators from the HEQED consortium, who were trained in the learning strategy, implemented the activities within their regular teaching sessions at their home institutions. Following the learning activity in class, the students were invited to complete a digital, anonymous survey.

In total, 264 students responded to the survey. They were enrolled at the bachelor- (90%), master (6%) or at the PhD-level (4%) either in the fields of health sciences, nursing, physiotherapy, social education, or sports- and health promotion. The distribution of study participants within our consortium were: University of Zaragoza (64%), Arcada University of Applied Sciences (21%), Western Norway University of Applied Sciences (13%) and HAN University of Applied Sciences (2%). The majority of participants (from the University of Zaragoza) worked on the learning activity “Maria and Alejandra”, while the participants from the other learning institutions worked on the “Dressing room”.

### Digital survey

The first part of the digital survey, using the software programme “Survey exact”, consisted of questions about the participants’ study field and level, and their university affiliation. Then, three closed and three open-ended questions were asked related to their experiences of taking part in the learning activity (see questions in Table 1). The questionnaire had been developed, tested and refined by the HEQED consortium, but were not further validated. The survey was distributed right after the learning activity, typically within the classroom setting, to capture their immediate responses. The survey was conducted in the period from November 2023 to May 2024.

**Table 1:** Overview of questions in the post learning activity survey

Question or statement	Response type
Understanding health equity is relevant for my studies/work life	5 point scale from strongly agree to strongly disagree
I want to learn more about health equity	5 point scale from strongly agree to strongly disagree
The learning experience was relevant for my studies/job	5 point scale from strongly agree to strongly disagree
Can you in short explain the learning activity you participated in?	Open-ended
Will you recommend others to be involved in a similar learning activity? Why/why not?	Open-ended
Do you have other comments to us?	Open-ended

### Analysis

#### Analysis of the closed questions

For the three closed-ended survey questions, response percentages were measured from the five-point scale, based on the total number of participants who completed the survey. No additional statistical analyses were conducted beyond these descriptive statistics since the quantitative data merely played a supportive role to the qualitative data material, which was issued for the main analysis for this paper. Thus, the descriptive statistics from the three closed questions (see the questions in Table 1) were included as additional elements to the thematic analysis of the open-ended responses.

#### Qualitative analysis of the open-ended questions

In our analysis of the open-ended responses, we were inspired by a reflexive thematic approach by Braun and Clarke [11, 12]. Throughout our analysis, we positioned ourselves as researchers with a particular attention on collaborative and co-constructed meaning-making. This meant that the analysis was conducted in multiple iterative phases within teams of researchers from the HEQED consortium. Initially, four sub-teams of researchers independently clustered and categorised student quotes and generated preliminary themes through manual coding. This process was inductive in nature, allowing themes to emerge organically from the data without applying a pre-existing coding frame. The next phase implied that the sub-teams collaboratively consolidated their preliminary themes, clusters, and illustrative quotes in an online workspace. Through a series of facilitated online discussions, the entire research team engaged in a reflexive dialogue to critically examine, refine, and negotiate the meaning of each theme. This process of collaborative sense-making aligns with the participatory orientation of our project and with the reflexive principles of thematic analysis, recognising researcher subjectivity as a resource rather than a bias to be controlled [11, 12]. Ultimately, this iterative and dialogic process led to the development of a set of shared and co-constructed themes, including sub themes and dimensions of those (see Table 2) that, we believe, reflect both the richness from the open-ended questions, and the collective interpretation carried out by the research team.

**Table 2:** Overview of themes, sub themes and dimensions of the sub themes

Theme	Sub theme and dimensions
Confronting cases	Real-life cases Experience bringing awareness Opening new views Various perspectives Real-world situations Close to reality Dilemmas Human rights and values Health equity and inequities Standing in ethical dilemmas Being confronted with injustices Challenging
Reflexive practise	Engagement Put ourselves in the shoes of others Debating, discussions, reflections Comparing opposite views of acting Eye-openers Social equity Broader understanding Awareness of prejudices Empathy Can be many solutions Health equity
Professional development	Urge to act Action of professionals Defending human rights Acting on injustices Standing and dealing with dilemmas Ethical responsibility Professional identity Taking different perspectives Taking action Working on complex/complicated issues Humanised as professionals Social knowledge and skills Professional development

### Ethical considerations

Participation in the project was based on informed consent. Information about the HEQED project, their participation, voluntariness and anonymity was given in both written and oral, typically in the classroom, prior to the learning activity. We underlined that no personally identifiable information would be collected, ensuring participant confidentiality, and that it was entirely voluntary to take part. The survey focused solely on participants’ learning experiences, posing minimal risk for the participants. Yet, we provided the students with contact details in case they needed a follow-up talk with their teacher from the HEQED consortium after participation.

## Results

Our exploration of the participants’ perceptions of the relevance, impact and pedagogic value of taking part in the intervention generated the following three main themes; 1) confronting cases, 2) reflexive practise, and 3) professional development. Each of those themes include two sub themes with underlying dimensions (Table 2). The descriptive analysis of the closed questions is added into those themes as informative, supportive elements.

### Confronting cases

*Confronting cases* concerns how students understand and interpret the pedagogy of the learning activity itself. The students emphasised particularly that it was a real-life case and that it provoked complex dilemmas. The theme reflects the participants’ perceptions of being challenged with a learning material that included real-life cases that prompted several dilemmas. From the closed-end questions, we learn that the great majority (95%) agreed or strongly agreed that the learning situations were relevant to their studies or work life. The participants expressed a connection to the cases provided because they were considered situations from the “real-world”. In the learning activity that we called “the dressing room”, were the students were challenged to add attributes to build a persona, the students actually developed their own case related to an hypothetical situation in the real world, as explained by a master student in health promotion from HAN University of Applied Sciences:

*“We were supposed to make a fictional character: define their age, sex and constitutional factors (such as ethnicity, gender, disabilities, etc.), their lifestyle (hobbies, lifestyles, norms and values, etc.), their social and community network and socioeconomic and cultural status”*

A student in the field of sports- and health promotion from Arcada University of Applied Sciences reflected upon “the dressing room” case by describing it as: *“It was educational and opened up a new perspective on how to view health”.*

The other case of our learning material, the story of “Alejandra and Maria” was tightly bound to reality in a professional social and healthcare setting. A student from the University of Zaragoza described the case as: *“We worked on a real situation of Alejandra and María.”* A classmate further explained that they were; *“working on the clinical case of a drug-addicted mother who gives birth to a daughter with NAS (Neonatal Abstinence Syndrome).* Apparently, this case was perceived as *“an activity that brings us closer to reality*”.

As the presentation of both cases was followed by discussions in smaller and larger groups in the learning situation, the participants were drawn into reflections about that truly provoked several dilemmas. The dilemmas became particularly evident when being challenged to take the perspective of various roles or *“stepping into the shoes”* of one or more of the characters in the case (e.g. the social worker or the mother). For instance, as stated by a nursing student from the University of Zaragoza: *“After the girl was born, social services took her to a foster family without consulting the mother”.* A running theme through the responses pointed towards the disrespected position of the mother in this case, which triggered awareness that;*“..allows you to expand your knowledge through real-life clinical cases”.*

According to our participants, this exercise of being confronted with a real-world case contributed to a better understanding of the situation as a whole as well as fostering empathy towards the various roles involved in a case. Several responses point to how they were confronted with injustice and how human rights were at stake, as exemplified by this statement from a nursing student from the University of Zaragoza:

*“We debated from different perspectives on the case of Maria and Alejandra, concluding that various human rights were violated. In Maria’s case, the early separation from her mother. Maria was not consulted or informed in advance about the decision, nor was she able to say goodbye to her daughter. In Vasile’s case, he was not given the right to even claim custody of his daughter.”*

The participants expressed how the confronting cases enabled them to consider viewpoints beyond their own cultural backgrounds in ways that encouraged open-mindedness around sensitive issues. This highlighted the human dimension of care and underscored the ethical responsibilities of health and social care professionals.

### Reflexive practise

Reflexive practises relate to how the learning situation triggered engagement in the group of students, especially through discussions about the cases. The case acted like a trigger towards involvement, as described by a student from the field of sports- and health promotion from Arcada University of Applied Sciences:: *“We identified the identities involved and then worked to improve their situation so that they could reach their full health potential.”* A nursing student from Spain expressed it as; “*We had to discuss what things we would have changed in the case of Alejandra and María, what things were done well, and what things were done poorly”.* Another nursing student from the University of Zaragoza pointed to; *“..and we discussed possible alternative solutions and how the characters acted”.*

The participants described how they engaged with the health equity case from a human rights perspective, seeing it from the standpoint of the different characters in the case and how the situation potentially could have had a different outcome if they had acted in a different way, as exemplified through this testimony from a nursing student from the University of Zaragoza:

*“A debate was held on a clinical case of Alejandra and María, considering the rights that were violated for each character, how they could have acted, and other possible outcomes”*.

The theme of reflexive practises was described as “*eye opening*” both regarding the inequities that occurred and to the role and possibilities of practitioners to act equitably, here expressed by a nursing student HAN University of Applied Sciences: *“It opens your eyes, you may think of something that you have not thought about before”.* The great majority (97%) of the participants agreed or strongly agreed that the topic of health equity is relevant for their job or their studies. They furthermore expressed their experience of the complexity of equitable care.

*“It is something that makes you think a lot, and there are several possible perspectives, making it almost impossible to take a stand on one opinion or another. I think it is something to consider” (nursing student from the University of Zaragoza)*

By engagement in groups around the cases, the reflections happened as part of a collaborative endeavour as a social educator student from Western Norway University of Applied Sciences wrote: *“Talking in groups and reflecting on challenges is an effective way to gain a deeper understanding of the different starting points people have”.* Although the open-ended nature of the learning activities was seen as beneficial by many, others expressed a preference for more structured guidance. A few participants found the expectation of active participation to be challenging. Some also shared that they struggled to express themselves freely and would have felt more comfortable if they had been assigned a role to play or given more time to prepare in advance.

### Professional development

The last theme, professional development, relates to an urge to act and to the development of a professional identity. The participants called for action through encouraging reflections on health equity issues. The great majority of students (90%) answered that they would like to learn more about health equity, but many students also underlined how the topic is of general concern in society. A student from HAN University of Applied Sciences expressed it like this: *“Everyone should know how society can be more open and accept each human being for who they are.”* In addition, our study participants questioned current practices for developing solutions that are fairer and more equitable. As a social educator student from Western Norway University of Applied Sciences commented: *“..people normally live and think within their own norms, and as we meet and work with people, we need to have a wider perspective to see and talk with people and the society”.* The change of actions is seen as a way of reaching more equitable care, but also for avoiding instrumentalization. A nursing student from the University of Zaragoza expressed it like this: *“We can change the protocols to avoid violating the human rights of both the parents and the child. With collaboration, we can improve the mother’s health and allow them to live together again”.*

A co-student stated that: *“..it puts us in a situation where we must question the actions of the participants, potentially identifying discrimination, protocol failures, coercive measures by healthcare staff, or even possible negligence”.*

The students expressed a strong commitment to using their knowledge and positions as social or health care professionals to critically evaluate protocols, and to make changes where equitable concerns are not addressed. The actions taken by social and healthcare professionals must be in the best interest of the citizen or the patient, calling for a “humanization of the protocols”. The students working on the *Dressing room* especially pointed to the need for creating holistic views, understanding the complexity in the different components affecting our health and well-being. A student from sports- and health promotion at Arcada University of Applied Sciences shared this: *“We learn from one another and develop a holistic understanding of people’s needs for an equitable and healthy way of life.”*

As described, the learning activities prompted reflections on equitable care and the responsibility to take action. The participants link this to their professional identity and how this might change in the future. They link their professional identity to the capability of acting based on the values/principles of health equity and to their own questioning of not only the protocols, but also the education. A nursing student from the University of Zaragoza expressed: *“We should not be satisfied with what is taught in class. We need to broaden our horizons to learn as much as possible”.* Several participants linked this learning to both their personal and professional lives, highlighting the close connection between personal life and professional identity. A nursing student from University of Zaragoza said that: “Thanks to these cases, we become more humanised as healthcare professionals, as our role is not only to care for health and be good professionals, but also as people, and empathy and humanization with others are very important”.

## Discussion

The results from our pilot study in the HEQED consortium responds to the European initiatives directed towards the critical concern about health equity in global health [25] by way of voicing how European students’ perceive the relevance, impact, and pedagogical value of health equity learning situations. Where higher education institutions are pointed out as a potential driver to fostering awareness and prepare students to act ethically and effectively in their future professional roles, our study offers intriguing insights directed towards students’ views towards learning about health equity in terms of confronting cases, reflexive practises and professional development. Foremost, these themes reveal a clear and widespread recognition of the relevance of health equity towards the academic and professional contexts of the students. Most students found the topic meaningful, and expressed a strong interest in learning more, and perceived the learning experience as valuable for their studies or future careers. This consistent pattern suggests that the pedagogical intervention aligned with students’ expectations and educational needs. Moreover, the high level of agreement across the responses survey questions highlights a shared understanding that of health equity should be as a central concern in global health education and affirms the importance of embedding such content meaningfully within higher education curricula. In the following discussion, we will in particular draw attention to the importance of encouraging critical reflection, and the transition from awareness to advocacy in the learning processes.

To discuss the issue of critical reflection in the learning process, we need to draw attention to the complex concept of health equity. It cannot be reduced to a single definition or a discrete learning outcome. It is inherently multifaceted and evolving, requiring a pedagogical approach that recognizes learning as a continuous, transformative process [15, 57]. Still, it has also been shown previously that the use of a compelling “trigger” case can be effective in anchoring this process, allowing students to engage both intellectually and emotionally with the structural determinants of health [6, 49]. From this pilot, we see that this case-based method can facilitate perspective-taking and encourage critical reflection on existing protocols. We know that this may support the development of empathy, which is an essential competency for equitable care [20]. The pedagogical approach adopted in this study aligns with the broader goal of fostering the ‘citizen scholar’, a graduate who is not only knowledgeable, but also ethically engaged and socially responsive [3]. The learning activity was explicitly framed around human rights and health equity, prompting students to identify rights violations and reflect on how professional actions can either undermine or promote equity. This framing enabled students to explore the values at stake in complex scenarios and to consider the ethical dimensions of professional practice. This reflection and increased awareness about one’s own biases are considered crucial for acting equitably in healthcare settings. It invites reflection on different perspectives and helps to question pre-established protocols that might contribute to inequity.

We need to draw upon Mezirow’s transformative learning theory (2000) when reflecting upon the transition from awareness about health equity to advocacy of health equity. The learning activity functioned as a catalyst for disorienting dilemmas, moments that challenged students’ assumptions and prompted deeper reflection [43]. These cognitive and emotional triggers were described by participants as “eye-opening” and “humanising”, suggesting that the activity succeeded in fostering epistemic awareness and ethical sensitivity. Such moments are foundational for developing a more equity-oriented professional identity [17, 27, 37]. Importantly, participants recognised that health equity is not solely the concern of social and healthcare professionals. The broader implications for fields such as law, education, and urban planning were also acknowledged, reinforcing the interdisciplinary relevance of the topic [38]. However, awareness alone is insufficient. As several scholars have noted, the transition from awareness to action requires the development of specific competencies, including advocacy, critical health literacy, and systems thinking [18, 46]. Emerging evidence points that these skills empower students to navigate and challenge the complex social and political structures that perpetuate inequities [28, 52]. Our findings identified several areas in which this tool fosters the development of critical knowledge, skills, and attitudes essential for professional growth. Learning about health equity is not a singular event, but may rather mark the beginning of a lifelong transformative journey, one that can be revisited and reinterpreted at different stages, particularly in professional practice [42, 54, 55].

The case-based, participatory approach was widely appreciated for its realism and relevance among the students in our pilot study. In line with Bruen et al. [19] the students valued the opportunity to engage with real-world scenarios, debate ethical dilemmas, and reflect on their own biases. The activity was described as both intellectually stimulating and emotionally impactful, contributing to both professional and personal growth. However, some participants found the open-ended nature of the task challenging. This can be seen as productive difficulty as learning theory suggests, cab support deeper engagement and conceptual transformation [21, 41]. Still, this discomfort highlights the need to scaffold such activities carefully. Feeling challenged can be a productive part of transformative learning, but it also requires a supportive environment where students feel safe to express themselves and explore difficult issues [53]. The suggestion to incorporate role-play was met with mixed responses. While some students appreciated the opportunity to “stand for” a position, others found it difficult to embody perspectives that conflicted with their own values. This tension underscores the importance of preparing students for responsible action in practice, encompassing ethical reasoning, contextual sensitivity, and, when necessary, advocacy, areas that traditional curricula often leave underdeveloped [55].

While this pilot study provides valuable insights into the use of equity-focused learning activities across diverse higher education contexts, several limitations must be acknowledged. The findings are based on self-reported data, which may be influenced by social desirability bias. Moreover, the absence of long-term follow-up restricts the ability to assess the sustained impact of the intervention or how students apply their learning in professional settings. While the activity aimed to promote equity in healthcare, it is also important to reflect on the equity of the learning environment itself (Ramdas et al. 2025). The study was implemented across multiple institutions, each adapting the learning activities to their own educational context. While this variation demonstrates the flexibility and relevance of the approach, it also limits the comparability of results and may affect generalisability. Students came from diverse academic backgrounds, learning institutions, and levels of study, which may have influenced their engagement and comfort with the material. Some participants reported difficulty expressing themselves or participating fully, particularly in group discussions. These dynamics underscore the need for inclusive facilitation strategies that account for differences in language, cultural background, and prior knowledge. Ensuring equitable participation is essential for realising the full potential of transformative learning ([27, 42], Massé et al. 2020; Ramdas et al. 2025).

The success of such pedagogical interventions also depends on the competencies and preparedness of educators. Facilitating discussions on ethically and emotionally charged topics requires not only subject expertise, but skills in managing group dynamics, supporting emotional safety, and guiding reflective dialogue. Institutions must therefore invest in training and supporting educators to adopt these roles effectively. Moreover, embedding health equity education across curricula requires institutional commitment to interdisciplinary collaboration and pedagogical innovation. Additionally, the survey was anonymous and did not collect sociodemographic data such as age, gender, making it difficult to explore differences in engagement or outcomes across student groups. Another limitation is the lack of information on how many students were invited to participate in the survey versus the number of respondents who actually participated. This prevents calculation of a response rate and limits the ability to assess the representativeness of the sample. Since the survey was not formally validated, that may affect the reliability of the findings.

Despite these limitations, the study offers a useful foundation for future research. Further development of the pedagogical model could include more structured role-play, follow-up sessions, and integration with broader curricular goals. Longitudinal and comparative studies across disciplines and educational levels would also enhance understanding of how different student populations engage with and benefit from health equity education.

## Conclusions

This pilot study demonstrates the potential of targeted pedagogical interventions to foster critical awareness, empathy and professional awareness concerning health equity among higher education students. Through the use of real-world, ethically complex cases, students engaged in reflective and transformative learning processes that challenged their assumptions and deepened their understanding of factors influencing health equity. The findings affirm that health equity is not only perceived as relevant across disciplines but also as essential to professional identity formation and ethical practice. The learning activity, grounded in human rights and participatory pedagogy, enabled students to explore the values and systemic structures that shape health outcomes. It also highlighted the importance of perspective-taking and critical reflection in preparing future professionals to act equitably. Future research should study the long-term impact of such interventions and explore how students translate their learning into professional practice. By integrating health equity education more systematically into higher education, institutions can make a meaningful contribution to the development of socially responsive graduates who are capable of advancing equity within and beyond the health sector.

## Supplementary Information

Supplementary Material 1.

## Data Availability

The datasets analysed during the current study are available from the corresponding author on reasonable request.

## Abbreviations

HEQED: Health Equity Through Education for a Sustainable Society
HVL: Western Norway University of Applied Sciences
NGO: Non-governmental organisation
PBL: Problem-based learning
UAS: University of Applied Sciences
