# Supplementary material for: Learning health equity in higher education—results from a European pilot study
Source: BMC Med Educ. 2026 Mar 11;26:632. doi: 10.1186/s12909-026-08979-1 (PMC13088603; doi:10.1186/s12909-026-08979-1)
Supplement: Supplementary file 1 — Supplementary Material 1. [file 12909_2026_8979_MOESM1_ESM.docx]

Supplementary material 1: Cases

More information can be found on: [www.heqed.org](https://www.heqed.org).

**Case: Maria and Alejandra**

The case of “Maria and Alejandra” is from a neonatal hospital unit, where we meet a birth-giving mother with her newborn daughter, as well as health- and social workers. Due to cocaine consumption during pregnancy, the baby got a withdrawal syndrome leading to social services abruptly taking the newborn in foster care. The students reflected on this case and the protocol that was followed through the lens of human rights.

Guidance was provided digitally through the HEQED Home (to be accessed via [www.heqed.org](https://www.heqed.org)). This is what they read:

*Background*

Baby Maria is admitted to LNU; Alejandra, her mother, accompanies her, never leaving her side and looking sad and concerned. Once they are both settled, the nurse responsible for Maria’s care asks her once again about substance consumption during her pregnancy. After much insistence on the nurse’s part, Alejandra finally admits that she has taken drugs in the past and that she used some about 20 days ago.

*Maria*

The information is passed on to the rest of the team and a urine drug screen test is done on baby Maria, which is positive to cocaine. Maria is diagnosed with neonatal abstinence syndrome (NAS) and is subsequently moved to a special area within LNU for further monitoring and assessment. In addition, her case is referred to the hospital’s social worker for evaluation by social services. Interventions after diagnosis include: Maintain a peaceful environment, low intensity light, etc. Exclusive artificial feeding; Alejandra must not breastfeed Maria. 50cc / 8 times / day. 8-hourly assessment of Maria’s NAS through the Finnegan Scale, designed to quantify the severity of NAS and guide treatment. Urine output and fluid balance (weigh nappies). Daily weight. Maria scores 6 on the Finnegan Scale and does not require medication (if 3 consecutive scores are equal to or greater than 8, treatment for withdrawal is started). She presents a light trembling episode and degree of hypertonia on day 2, which ceases spontaneously. By day 5 of her admission, Maria is feeding and resting adequately, and she is steadily gaining weight.

*Alejandra and Vasile*
Alejandra arrives every day at 6 am and stays with her daughter until 3 pm approximately; she then leaves to go to work and returns at midnight in time for her feed. Her attitude towards Maria is always loving and caring; she picks her up and asks frequently about her health. She tells the nurses that her whole family is terribly worried, and insists that she ”prays to God for Maria’s recovery”.

Vasile visits Maria every day at 6 pm. He feeds Maria but rarely interacts with her nor the staff. Alejandra frequently verbalizes her feelings and her concerns, especially at night. She does not want to lose her daughter and she tells the nurses that she fears Maria is going to be taken from her. She explains that her whole family is in Colombia and that she has no family in Spain that can look after the baby. More than anything, she does not want Vasile to have the custody of Maria.

*The hospital staff*

The hospital staff are kind to Alejandra, but they disapprove of her behavior. They are not surprised that she has been taking drugs; at the end of the day: "she is from Colombia". They comment that, although she looks genuinely worried about her daughter: "she should not have been taking drugs during her pregnancy in the first place"; “less praying to God and more taking good care of her daughter”.

*Social services and discharge*
After evaluation by social services, Maria’s custody was temporarily retired from Alejandra. This was not communicated to Alejandra nor to Vasile. The day that Maria was due to be discharged, two police officers, a social worker from the local government’s Child Protection Service, Maria’s foster parents and the hospital’s social worker turned up at the LNU. Alejandra was taken to a private interview room, where she was informed about the temporary withdrawal of Maria’s custody.

In the meantime, Maria was given to her foster family, who dressed and prepared her to leave the unit and take her home. When Alejandra came out from the interview room and saw that her child was no longer in her cot, she became very angry and cried, pleading with the staff not to take Maria away from her. The nursing staff and the social worker tried to calm her down and kindly invited her to leave the LNU immediately. That day, Alejandra left the LNU without Maria.

*Questions:*

1. Think about the story, what do you think? Perhaps more importantly, what do you feel?
2. How does health (in)equity impact on this story?

**Case: The dressing room**

The case of “the dressing room” presents a persona who initially is “undressed” in the sense of a number of variables, such as gender, age, nationality, family relations, language, interests, and other socio-economic, cultural and environmental background characteristics. The students’ task was to “dress up an identity” of the persona, (and also “re-dress” to reflect on what happens) and based upon that reflect jointly in groups and in plenum about how various conditions may contribute to health (in)equity.

Instructions can be provided by the teachers in the classroom or via digital instructions.

*Introduction and guidance*

The “Dressing room” is a pedagogical tool or concept for opening up discussion, thoughts and insights in health equity. The dressing room is a metaphor. The “dresses” are attributes, circumstances and conditions, and by changing them on a fictive persona/ avatar, the opportunities for health equity changes.

Link to video tutorial: [The dressing room](https://www.youtube.com/watch?v=7yZKtWS2ack)

[
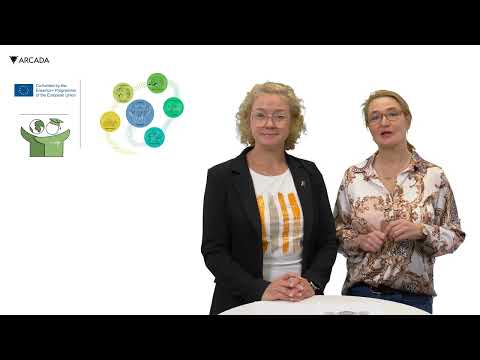
](https://www.youtube.com/watch?v=7yZKtWS2ack)
